# Children's behavioural and emotional wellbeing during the COVID-19 pandemic: Findings from the Born in Bradford COVID-19 mixed methods longitudinal study

**DOI:** 10.12688/wellcomeopenres.20752.1

**Published:** 2024-02-15

**Authors:** Ellena Badrick, Rachael H. Moss, Claire McIvor, Charlotte Endacott, Kirsty Crossley, Zahrah Tanveer, Kate E. Pickett, Rosemary R. C. McEachan, Josie Dickerson

**Affiliations:** 1 Bradford Institute for Health Research, Bradford Teaching Hospitals NHS Foundation Trust, Bradford, West Yorkshire, BD9 6RJ, UK; 2 Department of Health Sciences, University of York, York, England, UK

**Keywords:** COVID-19, mental health, children, poverty, health inequalities, ethnicity, social determinants of health, mixed methods, cohorts, Born in Bradford

## Abstract

**Background:**

The COVID-19 pandemic led to a multitude of immediate social restrictions for many across the world. In the UK, the lives of children and young people were quickly impacted when COVID-19 restrictions led to school closures for most children and restrictions on social interactions. The Born in Bradford COVID-19 longitudinal research study explored the impact of the COVID-19 pandemic on the lives of children and their families living in Bradford.

**Methods:**

Surveys were administered during the first wave of the pandemic (March to June 2020) and compared to findings from before the pandemic. The current study examined the social and emotional wellbeing of children from before to during the pandemic, measured using the parent completed Strengths and Difficulties questionnaire (SDQ). Regression analyses looked at associations between a range of social determinants of health and changes in SDQ scores.

**Results:**

The results showed that those children most likely to experience difficulties during the pandemic were boys, younger children, those from White British ethnicity (compared to Pakistani heritage children) and those living in the most deprived areas. There were associations between experiencing difficulties and: food insecurity; financial worry; getting below recommended levels of physical activity; and having less than the recommended amount of sleep.

**Conclusions:**

The effect of COVID-19 restrictions are likely to have had negative consequences on children that could, in time, have long-lasting impacts on the health, wellbeing and development of children in the UK.

## Plain Language Summary

The COVID-19 pandemic caused immediate and long-lasting social restrictions to be implemented here in the UK and across the world. In the UK, children and young people were quickly affected by these restrictions that led to school closures and other restrictions that prevented these individuals from socialising in person with one another. This study explored the impact that the pandemic had on the wellbeing of children by comparing data from before the pandemic with data collected during the pandemic. The data that has been collected looks at the behavioural strengths and difficulties that children are displaying. Our exploration found that children that were most likely to experience difficulties during the pandemic were boys, younger children, those who were White British and those who lived in the most deprived areas. The effect of the COVID-19 restrictions are likely to have had a negative impact on children and young people which in time may impact the health and development of children living here in the UK.

## Introduction

The lifestyle and routines of children across much of the world were disrupted as a consequence of COVID-19 associated restrictions in early 2020 ( ‘Childhood in the time of Covid’, 2020). In England, national measures used to reduce the spread of the virus (commonly referred to in the UK as ‘lockdown’) included school closures, restricted social interactions and a reduction in physical and leisure activities. These consequences have the potential for long-lasting impacts on the health and health-related behaviours of children and young people ( Bingham *et al.*, 2021; López-Bueno *et al.*, 2021).

A number of studies have reported negative effects on children’s health and wellbeing during the pandemic ( Cheng *et al.*, 2021; Whittle *et al.,* 2020). Lockdown measures increased feelings of loneliness, isolation, and depressive and anxiety symptoms in children and young people ( Hu & Qian, 2021; Lockyer *et al.*, 2022; Waite *et al.*, 2021). These findings are supported by Creswell *et al.* (2021), where results from a UK based longitudinal study using parent-reported measures of behavioural and attentional difficulties (Strengths and Difficulties questionnaire (SDQ)) showed clear increases during peak lockdown periods and when schools were closed. However, some studies reported minimal change in child wellbeing during the pandemic, suggesting that some children experienced little change to their wellbeing during the pandemic and that some became happier than they were prior to the start of the pandemic ( Cameron *et al.*, 2021; Gibson *et al.*, 2021; ImpactEd, 2022; Pybus *et al.*, 2022).

There is growing recognition that the pandemic restrictions could have increased health inequalities, for example, Bingham et al. found a significant reduction in the number of children achieving the recommended amount of physical activity during the first lockdown, which was strongly associated with ethnicity ( Bingham *et al.*, 2021). The Born in Bradford (BiB) COVID-19 Research Study was designed to understand the impact of COVID-19 restrictions on families with school-aged children living in the deprived and ethnically diverse city of Bradford ( Mceachan *et al.*, 2020). During this study, a range of validated questionnaires were completed by children including their socio-emotional wellbeing, measured using the SDQ. Prior to the pandemic, the same children and their parents had completed a similar questionnaire as a part of the BiB Growing up study ( Bird *et al.*, 2019). This provides a unique opportunity to understand which children were at most risk of poor health and wellbeing during the pandemic and if any key vulnerabilities were associated with a negative change in wellbeing.

The aim of this paper is to: 1) report children’s social and emotional wellbeing during the UK’s first COVID-19 lockdown and identify associated factors; 2) examine changes in children’s social and emotional wellbeing from before to during the first UK lockdown and identify any factors associated with a negative change in wellbeing; and 3) add insight to these findings through analysis of children’s main worries reported during the pandemic.

## Methods

### Setting

Situated in the North of England, Bradford is the 7th largest metropolitan district in England and Wales with a population of over 500,000 people (( Garlick, 2022), Office of National Statistics, 2022). Bradford has an ethnically diverse population with 57% identifying as White British origin, and 26% as having Pakistani heritage ( Office for National Statistics, 2021). Once a thriving industrial city, Bradford now has more households in deprivation than the average across England, with 57% of households in Bradford deprived in at least one dimension ( Office for National Statistics, 2021). The city also has higher than average levels of ill-health ( Mebrahtu *et al.*, 2015). The BiB research programme has been following the health and wellbeing of over 36,000 Bradford residents (adults and children) since 2007. It hosts three birth cohort studies with a focus on health inequalities in deprived and ethnic minority populations. The BiB family birth cohort recruited 12,453 mothers, 3,448 fathers, and 13,776 children between 2007–2011 ( Wright *et al.*, 2013). The most recent data capture pre-pandemic was the BiB Growing Up (BiBGU) and Primary School Years study which occurred between January 2017-March 2020, when children were aged between 6–11 years ( Bird *et al.*, 2019).

### Participants and data collection

The first of three BiB COVID-19 surveys was completed during the first wave of the pandemic which took place in March-June 2020; a total of 970 children participated. At this early stage of the pandemic, England had been put into the first lockdown, with the strictest conditions. Schools were closed, people were advised to work from home, stay home as much as possible and not mix with other households. This lockdown lasted several months, with only children of key workers and those with defined vulnerabilities able to attend school in person. It was early June 2020 when the restrictions began to ease, with reception, year 1 and year 6 children returning to school on a voluntary basis.

The pre-pandemic and pandemic surveys asked children to self-report on their physical activity, social activities and health. Parents/carers of respondents were also asked to complete the SDQ, reflecting on their child’s behaviour at this time point. The SDQ is a validated tool used to measure the social and emotional wellbeing of children and young people ( Goodman & Goodman, 2009). The SDQ parent version asks questions of parents/carers to assess the emotional symptoms, conduct problems, hyperactivity-inattention, peer problems and prosocial behaviour of their children. In the pandemic survey, children were also given free text questions that asked them to list “three things that you worry about at the moment” and “three things that make you feel happy/that you enjoy doing at the moment”. All surveys were completed and returned by post. Full details of the study methods are reported in the protocol paper ( McEachan *et al.*, 2020).

### Measures

***Outcome***

The total score of the SDQ was calculated from the subscales and dichotomised ( Dray *et al.*, 2016; Goodman & Goodman, 2009)) using a cut-off score of 14, with children who scored >=15 defined as experiencing difficulties in their social and emotional wellbeing ( Goodman & Goodman, 2009). The change in pre-pandemic and lockdown SDQ score was calculated in two ways. The first method dichotomised the scales and characterised those children who changed from experiencing no difficulties pre-pandemic to experiencing difficulties during the pandemic as one group. The second method kept the scales continuous and looked at the individuals change between the two time points.

***Covariates***

Demographic information: Sex, ethnicity and date of birth were taken from the baseline cohort data at the time of child’s birth (2007–2011) ( Wright *et al.*, 2013). Age was derived from date of birth and the COVID-19 questionnaire completion date. Ethnicity was recoded using the 2011 Census categories into a three-category variable representing the two largest ethnic groups in the city: Pakistani heritage and White British, as well as an ‘Other’ category to capture children from a wide range of additional ethnic groups within the sample.

Free school meals data were extracted from the BiB cohort routinely linked local authority data, with the most recent pre-pandemic information used in the analysis. Socioeconomic deprivation was measured by deriving quintiles of the 2019 Index of Multiple Deprivation (IMD) from the child’s address.

Modifiable risk factors: All variables were taken from the responses to the COVID-19 survey which used validated measures ( Bingham *et al.*, 2021; Mceachan *et al.*, 2020). Physical activity levels were measured by a modified version of the validated seven-day recall questionnaire, the Youth Activity Profile-English Youth Version. The variable ‘meeting physical activity guidelines’ was derived on whether children reported they had taken part in 60 minutes or more of moderate-to-vigorous physical activity on a usual weekday and weekend day in the previous week. Average hours of sleep categorised from normal bedtime and normal wake time responses. These were then categorised into three groups (<9hours (below recommended), 9-11 hours (recommended) and 11+hours (above recommended)).

Food insecurity was measured using the question “we can’t get the food we want because there is not enough money”, with answer options of ‘many times’, ‘1 or 2 times’ and ‘never’. Food insecurity was derived if children responded with ‘many times’ or ‘1 to 2 times’. Financial worry was measured using the question ‘I worry about how much money my family has’, with options of ‘yes’, ‘sometimes’, ‘never’, with ‘yes’ and ‘sometimes’ responses combined as denoting financial worry. We established those children still attending school by asking “Most of the schools across the country have now closed, and we want to find out about how you are now learning and doing work. Do you still go to school?” with responses ‘Yes’ or ‘No’.

### Data analysis

Quantitative survey data was analysed to identify the factors associated with difficulties on the SDQ during the pandemic, a multivariate logistic regression model was undertaken. Model 1 included the demographic variables of age, sex, IMD category, ethnic group and if the children were still attending school. Model 2 added the modifiable risk factors of food insecurity, financial insecurity, sleep (category) and physical activity (category).

To determine if there was a statistically significant difference in the number of children experiencing difficulties from before to during the pandemic, a McNemar’s test was completed on paired SDQ scores. A second multivariate logistic regression model was undertaken to identify factors associated with a negative change in SDQ score from before to during the pandemic. All statistical analyses were conducted in STATA, Version 17.

Qualitative responses to the open questions were analysed using thematic analysis and the dominant themes relating to behavioural and emotional wellbeing are described to help illuminate the findings from the quantitative data.

### Ethics statement

Ethical approval for BiBGU, including consent for this data to be used in other research studies was granted by Leeds Bradford Research ethics committee (reference: 16/YH/0320, 22/09/2016). Ethics to conduct COVID-19 surveys with children was granted via a substantial amendment (reference: 16/YH/0320), amendment 7, 31/03/2020). Parents gave informed consent for them and their child(ren) to participate in the BiB and BiBGU cohort study. For the COVID-19 survey, and as approved by the Health Research Authority and Bradford/Leeds research ethics committees, parents (and children) were sent an information sheet and completion of the questionnaire (via post or online) denoted consent implied consent all questionnaires completed.

### Patient and public involvement and engagement (PPIE) statement

Building on our long term relationships and commitment to PPIE within the BiB research programme we conducted a range of activities to inform our COVID-19 research ( Rahman *et al.*, 2022). These are outlined in McEachan *et al.* (2020). We reached out to a number of local community groups and stakeholders to gather ‘soft intelligence’ to inform research priorities. We used our existing parent and young ambassador advisory groups to help develop survey instruments and recruitment methods and help to interpret findings and disseminate these. We disseminated emerging findings in several ways to communities using a variety of platforms (e.g., WhatsApp, twitter, Facebook, newsletters).

## Results

### SDQ scores during the pandemic

Of the 970 children who completed the survey during lockdown, 851 had an SDQ completed by their parent/carer. Table 1 shows the characteristics of those children who completed the COVID-19 survey and had valid SDQ scores. Of those 439 (51.6%) were boys; 432 (52.2%) were of Pakistani heritage, 345 (41.7%) White British and 50 (6.2%) Other. The mean age at completion of the survey was 10.5 years. There were 321 (37.8%) children in the most deprived decile of IMD and 111 (13.8%) receiving free school meals. In our dataset only 19% of the white group are in the most deprived category. We observe the opposite for the South Asian population with 52.3% of the respondents from the most deprived areas. See supplementary Table 1 available as *Extended data* ( Dickerson, 2024). Table 2 shows the SDQ subscales and derived total SDQ scores, in this cohort 144 (16.9%) children would be classed as experiencing difficulties (scoring >14).

**Table 1.** Descriptive information for children who competed COVID-19 survey in March-June 2020 and had valid SDQ data available (N=851). SDQ, Strengths and Difficulties questionnaire; IMD, Index of Multiple Deprivation.

		Complete SDQ data	
		N	%
**Sex**	Boy	439	51.6
	Girl	412	48.4
**Age (**mean and SD)	Years	10.5	1.09
**Ethnicity**	White British	345	41.7
	South Asian	432	52.2
	Other	50	6.1
	Missing	24	
**IMD**	Deciles (most deprived)	321	37.7
	2 ^nd^ most	119	14.0
	3 ^rd^ most	146	17.2
	>4 ^th^ most	265	31.1
**Free School Meals**	No	695	86.2
	Yes	111	13.8
	Missing	45	
**Physical activity**	Below (0-30 mins)	229	34.6
	Normal (30-60 mins)	289	27.4
	Above (>=60 mins)	318	38.0
	Missing	15	
**Sleep**	mins	649.1	181.0
**Sleep**	Below (<9hours)	64	8.0
	Normal (9-11hours)	532	66.3
	Above (>11 hours)	206	27.5
	Missing	49	
**Food insecurity**	Yes	67	7.8
	Never	784	92.2
**Financial worry**	Yes	221	26.0
	No	630	74.0
**Attending School**	No	772	90.8
	Yes	79	9.2

**Table 2.** SDQ summary from responses in the COVID-19 survey. SDQ, Strengths and Difficulties questionnaire.

		N	
Completed all SDQ questions		851	
Emotional Score (mean SD)		2.02	2.25
Conduct Score (mean SD)		1.42	1.46
Hyperactivity Score (mean SD)		3.42	2.46
Peer Problems (mean SD)		1.91	1.71
Prosocial Score (mean SD)		8.06	1.94
Externalising Score (mean SD)		3.94	3.42
Internalising Score (mean SD)		4.85	3.58
Total difficulties score (mean SD)		8.06	1.94
Total difficulties score (n %)	Not experiencing difficulties (0-14)	707	83.1
	Experiencing difficulties (>=15)	144	16.9

Table 3 shows children’s sociodemographic details along with any potential risk factors for experiencing a higher SDQ score. The proportion of boys experiencing difficulties (20.5%) was greater than girls (13.1%), the most deprived group (23.1%) also experienced a higher proportion of difficulties compared to the least deprived group (12.5%). Those experiencing food insecurity also experienced more difficulties on the SDQ scale (40.3%) compared to those who did not (14.9%), and children who experienced financial worry had more difficulties (26.7%) than those who did not (13.2%). Children who had below recommended levels of physical activity (26.6%) compared to normal levels (15.2%) experienced more difficulties and for self-reported hours of sleep those getting below recommended levels (34.4%) experienced more difficulties compared to those who did not (14.5%).

**Table 3.** Risk factors by SDQ group of those completing the COVID-19 survey. SDQ, Strengths and Difficulties questionnaire; IMD, Index of Multiple Deprivation.

		Not experiencing difficulties	Experiencing difficulties	Total N
		%	%	
**Sex**	Boy	79.5	20.5	439
	Girl	86.9	13.1	412
**Ethnicity**	White British	80.3	19.7	345
	South Asian	83.8	16.2	432
	Other	90.0	10.0	50
**IMD**	Deciles (most deprived)	76.9	23.1	321
	2 ^nd^ most	90.8	9.2	119
	3 ^rd^ most	82.2	17.8	146
	>4 ^th^ most	87.6	12.5	265
**Attending school**	No	83.0	17.0	753
	Yes	83.5	16.5	79
**Food Insecurity**	No	85.1	14.9	784
	Yes	59.7	40.3	67
**Financial worry**	No	86.8	13.2	612
	Yes	73.3	26.7	221
**Physical activity**	Below (0-30 mins)	73.4	26.6	229
	Normal (30-60 mins)	84.8	15.2	289
	Above (>=60 mins)	88.0	12.0	318
**Sleep**	Below (<9hours)	65.6	34.4	64
	Normal (9-11hours)	85.5	14.5	532
	Above (>11 hours)	82.0	18.0	206

Table 4 shows the results of the multivariate analysis. In Model 1 we included non-modifiable risk factors. The data showed that the odds of children experiencing difficulties were three times greater if they lived in the most deprived quintile of IMD (OR 3.03, 95% CI 1.79-5.15). The odds of White British children experiencing difficulties were two times that of Pakistani heritage children (OR 0.55, 95% CI 0.35-0.86). Girls were less likely to experience difficulties compared to boys (OR 0.60, 95% CI 0.41-0.88), as were older, compared to younger children (per year: OR 0.81, 95% CI 0.68-0.96). There was no association between experiencing difficulties and whether the child continued to attend school during the pandemic.

**Table 4.** Multivariable model of demographic and modifiable risk factors associated with above cut point (poorer mental health) SDQ scores. SDQ, Strengths and Difficulties questionnaire; IMD, Index of Multiple Deprivation.

		Model 1			Model 2		
		Odds ratio	[95% conf. interval]		Odds ratio	[95% conf. interval]	
Age (increasing per year)		0.81	0.68	0.96	0.75	0.63	0.92
Sex	Boy	1.00			1.00		
	Girl	0.60	0.41	0.88	0.55	0.36	0.83
Ethnicity	White British	1.00			1.00		
	South Asian	0.55	0.35	0.86	0.47	0.29	0.75
	Other	0.34	0.13	0.92	0.24	0.08	0.73
IMD group	Deciles (most deprived)	3.03	1.79	5.15	2.03	1.15	3.62
	2nd most	0.97	0.45	2.10	0.86	0.38	1.94
	3rd most	1.91	1.04	3.50	1.54	0.81	2.97
	>4th most	1.00			1.00		
Attending school	No	1.00			1.00		
	Yes	0.81	0.42	1.55	0.94	0.47	1.90
Food Insecurity	No				1.00		
	Yes				3.27	1.71	6.23
Financial worry	No				1.00		
	Yes				1.90	1.20	3.01
Physical activity	Below				2.18	1.31	3.61
	Normal				1.00		
	Above				0.75	0.44	1.25
Sleep	Below				3.43	1.83	6.41
	Normal				1.00		
	Above				1.59	0.96	2.51

In a mutually adjusted Model 2 (with the addition of modifiable variables) there was no substantial difference to the relationships in Model 1, with those living in the most deprived areas (OR 3.03, 05% CI 1.79-5.15), those who are White British (OR 0.47 95% CI 0.29-0.75), boys (compared to girls, (OR 0.55, 95% CI 0.36-0.83) and children of a younger age (OR 0.75, 95% CI 0.63-0.92) continuing to be more likely to experience difficulties. In Model 2, the odds of experiencing difficulties were three times higher in those reporting food insecurity (OR 3.26, 95% CI 1.71-6.23), and almost two times higher in those experiencing financial worry (OR 1.90, 95% CI 1.19-3.01). Children with below recommended levels of physical activity had more than two times the odds of experiencing difficulties (OR 2.18, 95% CI 1.31-3.61) and children who had less than the recommended amount of sleep had more than three times the odds of experiencing difficulties (OR 3.43, 95% CI 1.83-6.41).

### Change in SDQ scores from pre-pandemic to during the pandemic

Pre-pandemic SDQ scores were available for 749 children (88% of those with COVID-19 survey SDQ scores, see Table 5). Using the same SDQ score cut point we saw 14.2% of children experienced difficulties pre-pandemic, compared to 17.6% during the pandemic. The majority of children’s SDQ scores were in the same category before and during the pandemic (n=656), with 34 (4.5%) children having a positive change (from experiencing difficulties to not) and 59 (8%) a negative change (from no reported difficulties to having difficulties). McNemar’s paired test was significant, indicating more children moved from not experiencing difficulties to experiencing difficulties during the first lockdown, compared to those experiencing difficulties at both time points. Proportion of children (8–12 years) reaching threshold for Mental Health Problems (using SDQ classification) by Ethnic Group and IMD category is shown in supplementary Graph 1 available as *Extended data* ( Dickerson, 2024). Using all respondent data (Total group on Graph 1 ( Dickerson, 2024)) we see a gradient via deprivation with more people in the most deprived group reaching the threshold, followed by the middle group, and those in the least deprived group had the smallest proportion of people reaching the SDQ threshold. All groups had an increase in proportion experiencing difficulties during the pandemic and the gradient was the same. We split the data by ethnic group into South Asian, White, and other (not shown on graph due to small numbers) and observed different patterns. The South Asian group showed the same pattern by deprivation, with an increased number during the pandemic for the most deprived group. The observation for the White group was distinctly different. We see those in the least deprived group experiencing more difficulties, and this increased during the pandemic.

**Table 5.** Factors influencing negative change in SDQ group compared to no change or positive change. Analysis limited to n=749 children who had pre-pandemic SDQ measured, 59 children had a negative change in SDQ score. Using a McNemar’s test of pre-pandemic SDQ score and COVID-19 SDQ score showed Chi2 6.721 and p=0.012 for a difference. SDQ, Strengths and Difficulties questionnaire; IMD, Index of Multiple Deprivation.

		Odds ratio	[95% conf. interval]		P-value
Age (increasing per year)		0.97	0.72	1.19	0.74
Sex	Boy	REF			
	Girl	0.51	0.32	0.83	0.07
Ethnicity	White British	REF			
	South Asian	0.42	0.25	0.72	0.001
	Other	0.30	0.10	1.02	0.053
IMD group	Most deprived	2.74	1.26	5.99	0.011
	2.00	0.76	0.24	2.37	0.637
	3.00	0.91	0.32	2.59	0.870
	>=4	REF			
Attending school	No	REF			
	Yes	0.65	0.28	1.48	0.308

A multivariable model was constructed to assess the factors associated with a negative change in SDQ score (those who had no difficulties pre-pandemic and moved to experiencing difficulties during the pandemic). The results of this analysis are shown in Table 5, and reflected those reported above, with girls (OR 0.51, 95% CI 0.32-0.83) and Pakistani heritage children (OR 0.42, 95% CI 0.25-0.72) less likely to experience a negative change in difficulties, and children living in the most deprived quintile more likely to experience a negative change in difficulties (OR 2.74, 95% CI 1.26-5.99). To explore this data further we calculated the change in SDQ score between the pre-pandemic and pandemic scores. We categorise any increase in difficulties score when children were found to experience more difficulties during the pandemic (compared to pre-pandemic) and these are shown in supplementary Table 2 available as *Extended data* ( Dickerson, 2024). This data was available for 749 individuals and n= 351 (46.9%) had an increase in SDQ score. The data did not however show any significant results.

### Qualitative analysis

From the 970 questionnaires completed, a total of 808 participants completed the free text question that asked children to report their three main worries. Two themes related to behavioural and emotional concerns were identified. The first was health anxiety relating to the COVID-19 virus with children reporting concerns about themselves or family members catching the virus and worrying that their parents/grandparents might die. Some children also reported broader worries such as ‘health’ or ‘family’. The second theme related to concerns about the children’s own mental health, with many reporting stress, anxiety, panic attacks and depression often related to the uncertainty and fear surrounding the virus and restrictions. Some expressed social anxiety concerns about friendships, experiencing negative emotions about themselves and bereavement. These themes are presented in Table 6.

**Table 6.** Key themes from free text analysis of children’s self-reported worries.

Theme	Description	Example quotes
**Theme 1:** Health Anxiety		
Catching virus (either themselves or loved ones)	Concerns about catching the virus, or family catching the virus. Worried about parents/grandparent dying. Sometimes participants will have just said ‘health’ or ‘family’.	“Someone dying”, “Spreading COVID-19”, “People I know getting coronavirus”
**Theme 2:** Mental Health		
Mental health	Experiencing stress, anxiety, panic attacks, depression	“When will things go back to normal”, “When can I go out without fear”, “When this pandemic will finish or if we will ever find a cure”

A total of 889 children completed the free text question which asked them to report up to three things that made them happy or that they enjoyed doing. No themes were identified that related directly to behavioural and emotional wellbeing; however, children did report a variety of activities that they enjoyed doing that may have helped to protect their behavioural and emotional wellbeing. The three most prominent themes within this free-text data are presented in Table 7.

**Table 7.** Key themes from free text analysis of things children reported enjoying or that made them happy.

Theme	Description	Example quotes
**Theme 1:** Digital Entertainment		
Gaming, online, TV	Enjoyed gaming (PS4, PC, XBOX etc.) often with friends online, social media (TikTok, Snapchat, YouTube etc.), watching TV and films	“Playing with friends online”, “Playing some video games with cousins”, “Watching movies”
**Theme 2:** Communicating with others		
Spending time with family	Report increased time spent with parents, siblings or other family members. Might just be reported as ‘family’	“Spending more time with my siblings”, “Staying safe with family”, “Spending time with my family more often than before”
**Theme 3:** Physical activity		
Being active/exercise	More time to be active/exercise has become more enjoyable/enjoying exercising with other family members (any physical activity, e.g., bike rides, runs, football, trampoline etc.)	“Going park with my family”, “Playing outdoors”, “Playing football in the garden”

## Discussion

### Principal Findings

Parents reported that 17% of children in our study experienced social and emotional wellbeing difficulties during the first COVID-19 lockdown. Compared to a pre-pandemic measure of SDQ, parents reported that 12.9% of children developed difficulties in their social and emotional wellbeing during the pandemic. Our results showed that children of a younger age, boys, those with White British ethnicity (compared to Pakistani ethnicity) and those living in the most deprived areas were more likely to experience difficulties during the UK’s first COVID-19 lockdown. In particular, children living in the most deprived areas were three times more likely to be experiencing difficulties. There was also an association between experiencing difficulties and food insecurity, financial worry, getting below recommended levels of physical activity and having less than the recommended amount of sleep.

The qualitative free text responses highlighted high levels of health anxiety, with responses detailing concern about catching COVID-19, spreading the virus and those they love becoming unwell or dying from the virus. Mentally, children were expressing high levels of worry and difficulties when adjusting to the pandemic rules, with many questioning when the pandemic would end and when ‘normality’ would return. However, responses also detailed things that children were enjoying which may have helped to protect them against negative changes during the pandemic including having time to use a game console and have more time with their family.

Other studies have similarly found that younger children and boys are more likely to experience difficulties ( Randall *et al.*, 2014). In our study, the mean age of the children was 10 years and the cohort included a mixture of primary and secondary school aged children. Younger children and boys might have experienced more negative impacts of the pandemic restrictions and/or anxieties relating to the virus itself, where older children may be better able to cope with the uncertainty. The finding that children of Pakistani heritage experienced less difficulties than White British children is interesting. In Bradford many Pakistani families live in intergenerational households, and children have, on average, more siblings than their White British peers. These living circumstances are believed to have increased the spread of, and persistence of, the COVID-19 virus within inner city areas of Bradford, however, they may also have provided a protective environment for children’s social and emotional wellbeing ( BIHR, 2022).

One of the key findings of this study is that child reported experience of food insecurity and financial worry during the pandemic was associated with the child experiencing more difficulties in their social and emotional wellbeing. Studies have acknowledged that children are at an increased risk of susceptibility to mental health difficulties and experienced substantial changes to their routines, physical and social isolation alongside high level of parental stress during the pandemic ( Imran *et al.*, 2020; Marques *et al.*, 2020). It was found that the area of deprivation was a highly influential factor in regard to whether or not a child was more likely to be experiencing difficulties. Those living in the most deprived areas were found to be three times more likely to be experiencing difficulties. Some studies have reported that vulnerable children may be less likely to ‘bounce back’ as restrictions eased ( Pybus *et al.*, 2022; Waite *et al.*, 2021). Other studies have shown that children identified as ‘at risk’ for mental health problems had increased levels of anxiety and depression if they lived in a household with parents and carers that experienced financial stress during the pandemic ( Cheng *et al.*, 2021; Ford *et al.*, 2021). Whilst the directions of association are not clear, interventions that enable children to have better sleep, be more physically active, and support families to be both financially and food secure will improve the social and emotional wellbeing of children.

### Strengths and limitations of the current study

Firstly, the study was conducted in a well-established cohort within an ethnically diverse and deprived population, which is not available elsewhere. Secondly, our study utilised the parent reported SDQ which may be better at reflecting accurately the child’s mental health. Thirdly, potential confounders were measured and included in the analysis. Finally, longitudinal data collection means we had pre-pandemic information to use as an individual comparator. There are limitations within the current study, firstly, the timing of the pandemic coinciding with the beginning of adolescence in this cohort, therefore the associations reported here may be reflective of wider social and personal changes irrespective of the pandemic. Secondly, although a wide array of methods were used to maximise survey response rates, engagement with the COVID-19 surveys was low and there may be some selection bias as children struggling with their mental health or family finances may have been more or less likely to complete the surveys. Thirdly, the survey measures are all self-reported and there could be bias in the reporting of e.g., activity levels. Fourthly, its possible unmeasured confounding variables may explain the results. However, comparing results with other studies of similar and differing populations will be important to gain a more detailed picture of the impact of the pandemic and its management on health and social inequalities ( Dickerson *et al.*, 2022).

## Conclusions

This study offers a unique assessment of the difficulties experienced by children in a highly ethnically diverse, seldom studied population, the majority of whom live in the most deprived centiles in the UK. The ongoing effects of the pandemic, particularly for the most disadvantaged, underscore the importance of recognising and meeting the support needs of children and families to ensure that inequalities are not widened further and children are given the opportunity to reach their full potential ( Creswell *et al.*, 2021). With ever increasing cost of living, energy prices and inflation since the pandemic, the ability of families to recover from the effects of the pandemic has been restricted. In case of any future pandemics, it is vital to consider how to better balance restrictions that limit the spread of a virus against the longer-term impact of any such restrictions on children’s social and emotional wellbeing.

### Next steps

In further research, it would be useful to identify whether the level of difficulties children experienced (at this time) improves, declines or stays the same as the pandemic progressed, and what factors (potentially protective) are different for those children who experienced fewer difficulties compared to those who experienced more difficulties. Targeted support for parents, carers or schools should be considered to provide individuals with the skills to feel better able to support their children in the aftermath of the COVID-19 pandemic (for example, mental health/physical activity check-ins, sleep hygiene sessions) and feel more able to help their children maintain positive mental health moving forwards.

## Ethics and consent

Ethical approval for BiBGU, including consent for this data to be used in other research studies was granted by Leeds Bradford Research ethics committee (reference: 16/YH/0320, 22/09/2016). Ethics to conduct COVID-19 surveys with children was granted via a substantial amendment (reference: 16/YH/0320), amendment 7, 31/03/2020). Parents gave informed consent for them and their child(ren) to participate in the BiB and BiBGU cohort study. For the COVID-19 survey, and as approved by the Health Research Authority and Bradford/Leeds research ethics committees, parents (and children) were sent an information sheet and completion of the questionnaire (via post or online) denoted consent implied consent all questionnaires completed.

## Data Availability

Researchers are encouraged to make use of the BiBBS data, which are available through a system of managed open access. Before you contact us, please make sure you have read our Guidance for Collaborators. Our BiB Executive reviews proposals on a monthly basis and we will endeavour to respond to your request as soon as possible. You can find out about the different datasets in our Data Dictionary. If you are unsure if we have the data that you need please contact a member of the BiB team ( borninbradford@bthft.nhs.uk). Once you have formulated your request please complete the ‘Expression of Interest’ form available here and send to borninbradford@bthft.nhs.uk. If your request is approved we will ask you to sign a Data Sharing Contract and a Data Sharing Agreement, and if your request involves biological samples we will ask you to complete a material transfer agreement. Harvard Dataverse: Children's behavioural and emotional wellbeing during the COVID-19 pandemic: Findings from the Born in Bradford COVID-19 mixed methods longitudinal study. https://doi.org/10.7910/DVN/FHOBDJ ( Dickerson, 2024). This project contains the following extended data: Extended data_Graph1.docx [Graph 1. Proportion of children (8–12 years) reaching threshold for mental health problems (using SDQ classification). Born in Bradford cohort and split by ethnic group and IMD category pre-pandemic and during the pandemic (March-June 2020)]. Extended data_Table1.docx [Extended data. Table 1. A breakdown of participants' ethnicity and IMD category]. Extended data_Table2.docx [Table 2. Factors influencing BiBGU participants' SDQ (reaching the threshold for social and emotional difficulties) from the BiBGU data (cross-sectional analysis), n=851. (2024-01-09)]. Data are available under the terms of the Creative Commons Zero "No rights reserved" data waiver (CC0 1.0 Public domain dedication).
