# Children's behavioural and emotional wellbeing during the COVID-19 pandemic: Findings from the Born in Bradford COVID-19 mixed methods longitudinal study

**DOI:** 10.12688/wellcomeopenres.20752.2

**Published:** 2025-09-03

**Authors:** Ellena Badrick, Rachael H. Moss, Claire McIvor, Charlotte Endacott, Kirsty Crossley, Zahrah Tanveer, Kate E. Pickett, Rosemary R. C. McEachan, Josie Dickerson

**Affiliations:** 1 Bradford Institute for Health Research, Bradford Teaching Hospitals NHS Foundation Trust, Bradford, West Yorkshire, BD9 6RJ, UK; 2 Department of Health Sciences, University of York, York, England, UK

**Keywords:** COVID-19, mental health, children, poverty, health inequalities, ethnicity, social determinants of health, mixed methods, cohorts, Born in Bradford

## Abstract

The COVID-19 pandemic led to a multitude of immediate social restrictions for many across the world. In the UK, the lives of children and young people were quickly impacted when COVID-19 restrictions led to school closures for most children and restrictions on social interactions. The Born in Bradford COVID-19 longitudinal research study explored the impact of the COVID-19 pandemic on the lives of children and their families living in Bradford. Surveys were administered during the first wave of the pandemic (March to June 2020) and compared to findings from before the pandemic (February 2017 to March 2020). The current study examined the social and emotional wellbeing of children ( *M* age: 10.5 years) from before to during the pandemic, measured using the parent completed Strengths and Difficulties Questionnaire (SDQ). Regression analyses looked at associations between a range of social determinants of health and changes in SDQ scores. The results showed that the odds of experiencing difficulties were lower for children of Pakistani heritage compared to White British children (OR 0.55, 95% CI 0.35-0.86). Girls were less likely to experience difficulties compared to boys (OR 0.60, 95% CI 0.41-0.88), as were older, compared to younger children (per year: OR 0.81, 95% CI 0.68-0.96) and those living in the most deprived quintile of IMD (OR 3.03, 95% CI 1.79-5.15). There were associations between experiencing difficulties and: food insecurity; financial worry; getting below recommended levels of physical activity; and having less than the recommended amount of sleep. These findings raise concerns about the longer-term impacts of the pandemic on children’s wellbeing and increasing inequity in child outcomes. The increased impact on the most disadvantaged, underscores the importance of recognising and meeting the support needs of children and families to ensure that inequalities are not widened further, and children are given the opportunity to reach their full potential.

## Introduction

The lifestyle and routines of children across much of the world were disrupted as a consequence of COVID-19 associated restrictions in early 2020 ( ‘Childhood in the time of Covid’, 2020). In England, national measures used to reduce the spread of the virus (commonly referred to as ‘lockdown’) included school closures, restricted social interactions, and a reduction in physical and leisure activities. There is clear evidence that these restrictions have had detrimental effects on a generation of young children across a wide range of outcomes, including educational, socioemotional, and physical wellbeing. Studies have reported developmental delay in young children ( Anand *et al.*, 2024), reduced physical activity ( Walker *et al.*, 2023), and increased mental ill health ( Hu & Qian, 2021; Lockyer *et al.*, 2022; Viner *et al.*, 2022; Waite *et al.*, 2021), leading some to view the pandemic as an adverse childhood experience, particularly in disadvantaged children.

The impact of these restrictions have the potential for long-lasting impacts on the behavioural and emotional health of children and young people ( Bingham *et al.*, 2021; Cheng *et al.*, 2021; López-Bueno *et al.*, 2021; Whittle *et al.*, 2020). There have been a large number of studies that have shown a significant increase in mental health problems of children and adolescents, particularly in girls, older children/adolescents, and in the first few months of the pandemic when the restrictions were most severe ( Miao *et al.*, 2023). Data from a UK based longitudinal study using the parent-reported SDQ showed clear increases in anxiety and depressive symptoms during peak lockdown periods ( Creswell *et al.*, 2021), compared to the level of anxiety and depressive symptoms pre-COVID-19.

However, other studies have reported minimal change in wellbeing during the pandemic, and some children reporting feeling happier than they were prior to the start of the pandemic ( Cameron *et al.*, 2021; Gibson *et al.*, 2021; ImpactEd, 2022; Pybus *et al.*, 2022). A recent paper using the Born in Bradford COVID-19 study data reported Pybus *et al.* (2022) that 55% of children reported no identifiable change in their wellbeing status from pre-pandemic to the first lockdown, and that children from a Pakistani background and those who reported peer issues prior to the pandemic, reported feeling less sad during lockdown.

These findings suggest potentially different experiences for differing groups of children. To explore this further, we analysed the SDQ data for children in the Born in Bradford (BiB) longitudinal COVID-19 Research to understand the impact of COVID-19 restrictions, and differences by ethnicity and socioeconomic circumstances, on children’s behaviour and emotional wellbeing ( Mceachan *et al.*, 2020).

Within the current study, we wanted to obtain further detail about the things that were worrying children at this time in the pandemic. We felt this would allow children to explain, in their words things that were on their mind at this time. It was hoped that this detail would help add context for us when understanding more about how the pandemic was impacting their wellbeing ( Dickerson *et al.*, 2016).

The aim of this paper was to: 1) report children’s social and emotional wellbeing, measured using the SDQ, during the UK’s first COVID-19 lockdown and identify associated factors in an ethnically diverse population; 2) examine changes in children’s social and emotional wellbeing from before the pandemic, to during the first UK lockdown, and identify social determinants associated with a negative change; and 3) add insight to these findings through analysis of free-text responses from children about their main worries during the pandemic.

## Methods

### Setting

Situated in the North of England, Bradford is the 7th largest metropolitan district in England and Wales with a population of over 500,000 people ( Garlick, 2022; Mebrahtu *et al.*, 2015). The BiB research programme has been following the health and wellbeing of over 36,000 Bradford residents (adults and children) since 2007. BiB was established in the city to identify the social determinants of poor health and wellbeing, in order to become a catalyst for change through the identification of modifiable causes of poor health and wellbeing, and the delivery of interventions to improve children’s life chances. The BiB family birth cohort recruited 12,453 mothers, 3,448 fathers, and 13,776 children between 2007-2011 ( Wright *et al.*, 2013). Just prior to the pandemic, the BiB Growing Up (BiBGU) study followed up children and their parents between February 2017-March 2020, when children were aged between 6–11 years ( Bird *et al.*, 2019).

### Participants and data collection

The BiB COVID-19 surveys were sent out to children who had taken part in the BiB Growing-Up study; a total of 970 children participated. All surveys were completed and returned by post. Full details of the study methods are reported in the protocol paper ( McEachan *et al.*, 2020).

The first survey was completed during the first wave of the pandemic between March–June 2020. This stage of the pandemic had the strictest conditions. Schools were closed, people were advised to work from home, stay home as much as possible and not mix with other households. This lockdown lasted several months, with only children of key workers and those with defined vulnerabilities able to attend school in person. It was early June 2020 when the restrictions began to ease, with reception, year 1 and year 6 children returning to school on a voluntary basis.

The BiB pre-pandemic and pandemic surveys asked children to report on their physical and mental health, social and online activities, what their main worries were, and what they enjoyed doing. Parents/carers of respondents were also asked to complete the SDQ. The SDQ parent version asks parents/carers to assess the emotional symptoms, conduct problems, hyperactivity-inattention, peer problems and prosocial behaviour of their children ( Goodman, 1997). Copies of the COVID-19 surveys administered can be found at https://borninbradford.nhs.uk/research/study-documents/.

### Measures

***Outcome***

The total score of the SDQ was calculated from the subscales and dichotomised ( Dray *et al.*, 2016; Goodman & Goodman, 2009) with children who scored >=15 defined as experiencing difficulties in their social and emotional wellbeing ( Goodman & Goodman, 2009). To understand changes in SDQ score from pre-pandemic to during pandemic, three categories were developed: 1) no change; 2) negative change (from no difficulty to experiencing difficulty); 3) positive change (from experiencing difficulty to no difficulty).

***Covariates***

Demographic information: Sex, ethnicity and date of birth were taken from the baseline cohort data at the time of child’s birth (2007–2011) ( Wright *et al.*, 2013). Age was derived from date of birth and the COVID-19 questionnaire completion date. Ethnicity was recoded using the 2011 Census categories into a three-category variable representing the two largest ethnic groups in the city: Pakistani heritage and White British, as well as an ‘Other’ category to capture children from a wide range of additional ethnic groups within the sample. Free school meals data were extracted from the BiB cohort routinely linked local authority data, with the most recent pre-pandemic information used in the analysis. Socioeconomic deprivation was measured by deriving quintiles of the 2019 Index of Multiple Deprivation (IMD) from the child’s address.

Modifiable risk factors: All variables were taken from the responses to the COVID-19 survey ( Bingham *et al.*, 2021; Mceachan *et al.*, 2020). Physical activity levels were measured using the Youth Activity Profile-English Youth Version. Meeting physical activity guidelines was 60 minutes or more of moderate-to-vigorous physical activity on a usual weekday and weekend day. Average hours of sleep categorised from normal bedtime and normal wake time responses. These were then categorised into three groups (<9hours (below recommended), 9–11 hours (recommended) and 11+hours (above recommended)). This data was captured using a modified version of the Youth Activity profile ( Fairclough *et al.*, 2019).

Food insecurity was measured using the question “we can’t get the food we want because there is not enough money”, children were classified as food insecure if they responded with ‘many times’ or ‘1 to 2 times’ ( Fram *et al.*, 2013). Financial worry was measured using the question ‘I worry about how much money my family has’, with options of ‘yes’ and ‘sometimes’ responses combined as denoting financial worry. We established those children still attending school by asking “Most of the schools across the country have now closed, and we want to find out about how you are now learning and doing work. Do you still go to school?” with responses ‘Yes’ or ‘No’.

Children’s worries and things that they were enjoying were measured by using the following questions: i) What are your three biggest worries right now? and ii) Has lockdown made any parts of life easier or more enjoyable?

### Data analysis

To identify the factors associated with difficulties on the SDQ during the pandemic, a multivariate logistic regression model was undertaken. Model 1 included the demographic variables of age, sex, IMD category, ethnic group and if the children were still attending school. Model 2 added the modifiable risk factors of food insecurity, financial insecurity, sleep (category) and physical activity (category).

To determine if there was a statistically significant difference in the number of children experiencing difficulties from before to during the pandemic, a McNemar’s test was completed on paired SDQ scores. A second multivariate logistic regression model was undertaken to identify factors associated with a negative change in SDQ score from before to during the pandemic. All statistical analyses were conducted in STATA, Version 17.

Qualitative responses to the free text questions were analysed by four authors (RM, CE, ZT, BG) using thematic analysis. Analysis was initially conducted independently and then proposed codes were discussed by all authors (RM, CE, ZT, BG) until agreement on a suitable codebook could be reached. Once a codebook (with suitable codes and definitions) had been created, data was independently analysed using this codebook. Any discrepancies in coding were discussed until agreement (between both authors) was reached. Any final changes that were needed to be made to the codebook were made and a subsequent summary of identified codes was written. In this paper, the themes relating to behavioural and emotional wellbeing are described to help illuminate the findings from the quantitative data.

### Ethics statement

This study involves human participants and ethical approval was obtained from the National Health Service Health Research Authority Yorkshire and the Humber (Bradford Leeds) Research Ethics Committee). Ethical approval to conduct COVID-19 surveys was granted by Leeds Bradford Research ethics committee via a substantial amendment (reference: 16/YH/ 0062, substantial amendment to BiBGU 16/YH/0320). Parents gave informed consent for them and their child(ren) to participate in the BiB and BiBGU cohort study. For the COVID-19 survey, and as approved by the Health Research Authority and Bradford/Leeds research ethics committees, parents (and children) were sent an information sheet and completion of the questionnaire (via post) denoted implied consent.

### PPIE statement

Building on our long term relationships and commitment to PPIE within the BiB research programme we conducted a range of activities to inform our COVID-19 research ( Rahman *et al.*, 2022). These are outlined in McEachan *et al.* (2020). We reached out to a number of local community groups and stakeholders to gather ‘soft intelligence’ to inform research priorities. We used our existing parent and young ambassador advisory groups to help develop and test the survey instruments and recruitment methods and help to interpret findings and disseminate these. We disseminated emerging findings in several ways to communities using a variety of platforms (e.g., WhatsApp, twitter, Facebook, newsletters).

## Results

### SDQ scores during the pandemic

Of the 970 children who completed the COVID-19 survey, 851 had a SDQ completed by their parent/carer. Table 1 shows the characteristics of those children who completed the COVID-19 survey and had valid SDQ scores. Of those 439 (51.6%) were boys; 432 (52.2%) were of Pakistani heritage, 345 (41.7%) White British and 50 (6.2%) Other. The mean age at completion of the survey was 10.5 years. There were 321 (37.8%) children in the most deprived decile of IMD and 111 (13.8%) receiving free school meals. Table 2 shows the SDQ subscales and derived total SDQ scores. In this cohort, 144 (16.9%) children scored >=15, defined as experiencing difficulties.

**Table 1.** Descriptive information for children who competed COVID-19 survey in March–June 2020 and had valid SDQ data available (N=851).

		Complete SDQ data	
		N	%
**Gender**	Boy	439	51.6
	Girl	412	48.4
**Age**	Years (mean and SD)	10.5	1.09
**Ethnicity**	White British	345	41.7
	Pakistani	432	52.2
	Other	50	6.1
	Missing	24	
**IMD**	Deciles (most deprived)	321	37.7
	2 ^nd^ most	119	14.0
	3 ^rd^ most	146	17.2
	>4 ^th^ most	265	31.1
**Free School Meals**	No	695	86.2
	Yes	111	13.8
	Missing	45	
**Physical activity**	Below (0–30 mins)	229	34.6
	Normal (30–60 mins)	289	27.4
	Above (>=60 mins)	318	38.0
	Missing	15	
**Sleep#**	mins	649.1	181.0
**Sleep**	Below (<9hours)	64	8.0
	Normal (9–11hours)	532	66.3
	Above (>11 hours)	206	27.5
	Missing	49	
**Food insecurity**	Yes	67	7.8
	Never	784	92.2
**Financial worry**	Yes	221	26.0
	No	630	74.0
**Attending School**	No	772	90.8
	Yes	79	9.2

**Table 2.** SDQ summary from responses at in the COVID-19 survey, and in the pre-pandemic survey.

		N		N	
Completed all SDQ questions		851		749	
Emotional Score (mean SD)		2.02	2.25	2.20	2.21
Conduct Score (mean SD)		1.42	1.46	1.47	1.58
Hyperactivity Score(mean SD)		3.42	2.46	3.32	2.51
Peer Problems (mean SD)		1.91	1.71	1.53	1.68
Prosocial Score(mean SD)		8.06	1.94	8.68	1.80
Externalising Score (mean SD)		3.94	3.42	4.80	3.57
Internalising Score (mean SD)		4.85	3.58	3.73	3.26
Total difficulties score (mean SD)		8.06	1.94	8.52	5.87
Total difficulties score (n %)	Not experiencing difficulties (0–14)	707	83.1	738	85.7
	Experiencing difficulties (>=15)	144	16.9	123	14.3

To see details on change in SDQ scores please see supplementary Table 2.

Table 3 shows the SDQ scores alongside the children’s sociodemographic details. The proportion of boys experiencing difficulties (20.5%) was greater than girls (13.1%), the most deprived group (23.1%) experienced a higher proportion of difficulties compared to the least deprived group (12.5%). Those experiencing food insecurity also experienced more difficulties on the SDQ scale (40.3%) compared to those who did not (14.9%), and children who experienced financial worry had more difficulties (26.7%) than those who did not (13.2%). Children who had below recommended levels of physical activity (26.6%) compared to normal levels (15.2%) experienced more difficulties and for self-reported hours of sleep those getting below recommended levels (34.4%) experienced more difficulties compared to those who did not (14.5%).

**Table 3.** Risk factors by SDQ group COVID-19 survey.

		Not experiencing difficulties	Experiencing difficulties	Total N
		%	%	
**Sex**	Boy	79.5	20.5	439
	Girl	86.9	13.1	412
**Ethnicity**	White British	80.3	19.7	345
	Pakistani	83.8	16.2	432
	Other	90.0	10.0	50
**IMD**	Deciles (most deprived)	76.9	23.1	321
	2 ^nd^ most	90.8	9.2	119
	3 ^rd^ most	82.2	17.8	146
	>4 ^th^ most	87.6	12.5	265
**Attending school**	No	83.0	17.0	753
	Yes	83.5	16.5	79
**Food Insecurity**	No	85.1	14.9	784
	Yes	59.7	40.3	67
**Financial worry**	No	86.8	13.2	612
	Yes	73.3	26.7	221
**Physical activity**	Below (0–30 mins)	73.4	26.6	229
	Normal (30–60 mins)	84.8	15.2	289
	Above (>=60 mins)	88.0	12.0	318
**Sleep**	Below (<9hours)	65.6	34.4	64
	Normal (9–11hours)	85.5	14.5	532
	Above (>11 hours)	82.0	18.0	206

Table 4 shows the results of the multivariate analysis. In Model 1 we included non-modifiable risk factors. The data showed that the odds of children experiencing difficulties were three times greater if they lived in the most deprived quintile of IMD (OR 3.03, 95% CI 1.79-5.15). The odds of experiencing difficulties were lower for children of Pakistani heritage compared to White British children (OR 0.55, 95% CI 0.35-0.86). Girls were less likely to experience difficulties compared to boys (OR 0.60, 95% CI 0.41-0.88), as were older, compared to younger children (per year: OR 0.81, 95% CI 0.68-0.96). There was no association between experiencing difficulties and whether the child continued to attend school during the pandemic.

**Table 4.** Multivariable model of demographic and modifiable risk factors associated with above cut point (poorer mental health) SDQ scores.

		Mode 1			Model 2		
		Odds ratio	[95% conf. interval]		Odds ratio	[95% conf. interval]	
Age (increasing per year)		0.81	0.68	0.96	0.75	0.63	0.92
Sex	Boy	1.00			1.00		
	Girl	0.60	0.41	0.88	0.55	0.36	0.83
Ethnicity	White British	1.00			1.00		
	Pakistani	0.55	0.35	0.86	0.47	0.29	0.75
	Other	0.34	0.13	0.92	0.24	0.08	0.73
IMD group	Deciles (most deprived)	3.03	1.79	5.15	2.03	1.15	3.62
	2 ^nd^ most	0.97	0.45	2.10	0.86	0.38	1.94
	3 ^rd^ most	1.91	1.04	3.50	1.54	0.81	2.97
	>4 ^th^ most	1.00			1.00		
Attending school	No	1.00			1.00		
	Yes	0.81	0.42	1.55	0.94	0.47	1.90
Food Insecurity	No				1.00		
	Yes				3.27	1.71	6.23
Financial worry	No				1.00		
	Yes				1.90	1.20	3.01
Physical activity	Below				2.18	1.31	3.61
	Normal				1.00		
	Above				0.75	0.44	1.25
Sleep	Below				3.43	1.83	6.41
	Normal				1.00		
	Above				1.59	0.96	2.51

In a mutually adjusted Model 2, with the addition of modifiable variables there was no substantial difference to the sociodemographic relationships found in Model 1, with those living in the most deprived areas (OR 3.03, 05% CI 1.79-5.15), those who are White British (compared to Pakistani heritage (OR 0.47 95% CI 0.29-0.75)), boys (compared to girls, (OR 0.55, 95% CI 0.36-0.83)) and children of a younger age (OR 0.75, 95% CI 0.63-0.92) continuing to be more likely to experience difficulties. In this model, the odds of experiencing difficulties were three times higher in those reporting food insecurity (OR 3.26, 95% CI 1.71-6.23), and almost two times higher in those experiencing financial worry (OR 1.90, 95% CI 1.19-3.01). Children with below recommended levels of physical activity had more than two times the odds of experiencing difficulties (OR 2.18, 95% CI 1.31-3.61) and children who had less than the recommended amount of sleep had more than three times the odds of experiencing difficulties (OR 3.43, 95% CI 1.83-6.41).

### Change in SDQ scores from pre-pandemic to during the pandemic

Pre-pandemic SDQ scores were available for 749 children (88% of those with COVID-19 survey SDQ scores, see Table 5). In this sample, 14.2% of children experienced difficulties pre-pandemic, compared to 17.6% during the pandemic. The majority of children’s SDQ scores were categorised as having no change (staying in the same category) before and during the pandemic (n=656). 34 (4.5%) children had a positive change (from experiencing difficulties to not) and 59 (8%) a negative change (from no reported difficulties to having difficulties). McNemar’s paired test was significant (Chi2 6.721, p=0.012), indicating more children moved from not experiencing difficulties to experiencing difficulties during the first lockdown, compared to those experiencing difficulties at both time points.

**Table 5.** Factors influencing negative change in SDQ group compared to no change or positive change. Analysis limited to n= 749 children who had pre-pandemic SDQ measured, 59 children had a negative change in SDQ score. Using a McNemar’s test of pre-pandemic SDQ score and COVID-19 SDQ score showed Chi2 6.721 and p=0.012 for a difference.

		Odds ratio	[95% conf. interval]		P-value
Age (increasing per year)		0.97	0.72	1.19	0.74
Sex	Boy	REF			
	Girl	0.51	0.32	0.83	0.07
Ethnicity	White British	REF			
	Pakistani	0.42	0.25	0.72	0.001
	Other	0.30	0.10	1.02	0.053
IMD group	Most deprived	2.74	1.26	5.99	0.011
	2.00	0.76	0.24	2.37	0.637
	3.00	0.91	0.32	2.59	0.870
	>=4	REF			
Attending school	No	REF			
	Yes	0.65	0.28	1.48	0.308

A multivariable model was constructed to assess the sociodemographic factors associated with a negative change in SDQ score (those who had no difficulties pre-pandemic and moved to experiencing difficulties during the pandemic. The results of this analysis, shown in Table 5, reflected those reported in the previous analyses, with girls (compared to boys (OR 0.51, 95% CI 0.32-0.83)) and Pakistani heritage children (compared to White British (OR 0.42, 95% CI 0.25-0.72)) being less likely to experience a negative change in difficulties, and children living in the most deprived quintile more likely to experience a negative change in difficulties (OR 2.74, 95% CI 1.26-5.99).

### Qualitative analysis

From the 970 questionnaires completed, a total of 808 participants completed the free text question that asked children to report their three main worries. There were two themes that related to behavioural and emotional concerns. The first was health anxiety relating to the COVID-19 virus with children reporting concerns about themselves or family members catching the virus and worrying that their parents/grandparents might die.

“Someone dying”

 “Spreading COVID-19”

“People I know getting coronavirus”

The second theme related to the fear and uncertainty surrounding the virus and restrictions. This led some children to report mental health related symptoms that they were experiencing (e.g., stress, anxiety and fear).

“When will things go back to normal”

“When can I go out without fear”

“When this pandemic will finish or if we will ever find a cure”

Some expressed social anxiety concerns about friendships, experiencing negative emotions about themselves and bereavement.

889 children completed the free text question which asked them to report three things that made them happy or that they enjoyed doing. No themes were identified that related directly to behavioural and emotional wellbeing, however, children did report a variety of activities that they enjoyed doing, including playing with their friends online, playing out in their own gardens, and spending time with their family, that may have helped to protect their behavioural and emotional wellbeing.

“Playing with friends online”,

“Playing football in the garden”

“Spending time with my family more often than before”

## Discussion

### Principal findings

During the first COVID-19 lockdown in the UK, 17% of children in the BiB COVID-19 survey experienced social and emotional wellbeing difficulties. Our results showed that children of a younger age, boys, those with White British ethnicity (compared to Pakistani ethnicity) and those living in the most deprived areas were more likely to experience difficulties during the first COVID-19 lockdown. There was also an association between experiencing difficulties and food insecurity, financial worry, getting below recommended levels of physical activity and having less than the recommended amount of sleep. This finding is similar to the work of Holst *et al.* (2023) where they identified that young people with difficulties, specifically, certain educational needs and/or neurodevelopmental disorders were more likely to show high (albeit stable) or increasing levels of mental health difficulties ( Guzman Holst *et al.*, 2023). Compared to the pre-pandemic measure of SDQ, 12.9% of children developed new difficulties in their social and emotional wellbeing during the pandemic. This was more common in boys, White British and older age groups. The free text responses highlighted high levels of health anxiety in children for themselves and their family related to the COVID-19 virus, and fear and uncertainty about what was going to happen which may have caused some of the increased emotional changes in SDQ scores during the pandemic.

### Strengths and limitations of the current study

This study was conducted in a well-established longitudinal cohort within an ethnically diverse and deprived population, who are often underrepresented in research, and was able to utilise pre-pandemic SDQ scores as an individual baseline comparator, which is not available in other studies. The study utilised the validated parent reported SDQ which accurately reflects child behavioural and emotional difficulties. There are limitations within the current study. Firstly, the timing of the pandemic coincided with the beginning of adolescence for some children in this cohort, therefore the associations reported here may be reflective of wider social and personal changes irrespective of the pandemic. Secondly, although a wide array of methods were used to maximise survey response rates, engagement with the COVID-19 surveys was low and there may be some selection bias as children struggling with their mental health or family circumstances may have been more or less likely to complete the surveys ( Mceachan *et al.*, 2020). Thirdly, the survey measures are all self-reported and there could be bias in the reporting of e.g., activity levels. In addition, we used parent reported SDQ data and this could be skewed by reporting bias if the parents experience of the pandemic was particularly difficult at that time. Fourthly, it’s possible unmeasured confounding variables may explain the results.

### Wider context

Other studies have similarly found that younger children and boys are more likely to experience emotional difficulties during the pandemic ( Randall *et al.*, 2014). Together, these findings suggest that younger children, and boys in particular, may need better information / communication tools to explain and support them during times of major events such as the pandemic. The importance of physical activity and sleep in maintaining health socioemotional wellbeing should also be clearly communicated, with suggested activities to improve these behaviours in times of severe restrictions, particularly among socio-economic deprived children. We did not separate the data by primary and secondary age children, and it is possible an interaction between experience of lockdown, school closures and level of academic provision at the school level may have an impact.

The finding that children of Pakistani heritage, in the Bradford study, experienced less difficulties that White British children is not unique to this study, and highlights the potentially protective effects of different familial environments. In Bradford, many Pakistani families live in intergenerational households, and children have, on average, more siblings than their White British peers. These living circumstances are believed to have increased the spread of, and persistence of, the COVID-19 virus within inner city areas of Bradford, however, they appear to have also provided a protective environment for children’s social and emotional wellbeing ( BIHR, 2022).

One of the key findings of this study is that children who reported food insecurity and financial worry during the pandemic was associated with the child experiencing more difficulties in their social and emotional wellbeing ( Imran *et al.*, 2020; Marques *et al.*, 2020). We also found that those living in the most deprived areas had three times the odds of experiencing difficulties. This has been reported in other UK studies, increased financial insecurity and time spent on childcare and home schooling were related to a deterioration of mental health (worse for working parents). This burden was not shared equally between men and women, and between richer and poorer households ( Cheng *et al.*, 2021). Furthermore, the pandemic has been shown to have increased financial insecurity and pushed many more families into poverty, thereby further increasing the number of children at risk of poor behavioural and socio-emotional outcomes. Financial support and protection for families is key, and more should be done to protect families from food and financial insecurity in times of social restrictions. These inequalities ought to be considered when crafting policy responses.

As a result of school closures and strict restrictions regarding going outside home, children have been one of the most disadvantaged groups over the extended pandemic period which may have contributed to a ‘double disadvantage’ for these children as they miss out on interactions with friends but also health benefits of physical activity in schools and provision of food ( López-Bueno *et al.*, 2021; Viner *et al.*, 2022). Future studies need to consider short and long-term sequels of social isolation.

### What does this study offer?

This study offers a unique assessment of the difficulties experienced by children in a highly ethnically diverse, seldom studied population, the majority of whom live in the most deprived centiles in the UK. However, comparing results with other studies of similar and differing populations will be important to gain a more detailed picture of the impact of the pandemic and its management on health and social inequalities ( Dickerson *et al.*, 2022). The ongoing effects of the pandemic, particularly for the most disadvantaged, underscore the importance of recognising and meeting the support needs of children and families to ensure that inequalities are not widened further and children are given the opportunity to reach their full potential ( Creswell *et al.*, 2021).

### Future research

It would be useful to identify whether the level of difficulties children experienced changed as the pandemic progressed, and what factors (potentially protective) are different for those children who experienced fewer difficulties. Our aim was to establish what sociodemographic factors were associated with poorer mental health in children, future studies to look at modifiable factors and changes in family circumstances during the COVID-19 pandemic will be important to contextualise our findings. Targeted support for parents, carers or schools should be considered to provide individuals with the skills to feel better able to support their children in the aftermath of the COVID-19 pandemic (for example, mental health/physical activity check-ins, sleep hygiene sessions). Longer term impact of distance learning on educational attainment and if critical periods can be identified to aid enhance support e.g., those at the transition from primary to secondary school. In case of any future pandemics, it is vital to consider how to better balance restrictions that limit the spread of a virus against the longer-term impact of any such restrictions on children’s social and emotional wellbeing.

## Figures and Tables

**Table S1.** 

		Model 1			Model 2		
		Odds ratio	[95% conf. interval]		Odds ratio	[95% conf. interval]	
Age (increasing per year)		0.92	0.80	1.06	0.92	0.79	1.07
Sex	Boy	1.00			1.00		
	Girl	1.06	0.78	1.44	1.08	0.79	1.49
Ethnicity	White British	1.00			1.00		
	Pakistani	1.02	0.70	1.47	0.98	0.66	1.45
	Other	1.16	0.60	2.24	1.19	0.60	2.36
IMD group	Deciles (most deprived)	0.61	0.38	1.01	0.73	0.44	1.21
	2 ^nd^ most	0.91	0.58	1.40	1.01	0.64	1.59
	3 ^rd^ most	0.97	0.64	1.47	1.14	0.73	1.76
	>4 ^th^ most	1.00			1.00		
Attending school	No	1.00			1.00		
	Yes	1.15	0.70	1.90	1.31	0.78	2.22
Food Insecurity	No				1.00		
	Yes				1.32	0.73	2.41
Financial worry	No				1.00		
	Yes				1.11	0.78	1.58
Physical activity	Below				1.23	0.82	1.82
	Normal				1.00		
	Above				0.80	0.55	1.16
Sleep	Below				1.49	0.85	2.64
	Normal				1.00		
	Above				1.04	0.71	1.52
