# Supplementary material for: Children's behavioural and emotional wellbeing during the COVID-19 pandemic: Findings from the Born in Bradford COVID-19 mixed methods longitudinal study
Source: Wellcome Open Res. 2025 Sep 3;9:36. Originally published 2024 Feb 15. [Version 2] doi: 10.12688/wellcomeopenres.20752.2 (PMC11109562; doi:10.12688/wellcomeopenres.20752.2)
Supplement: Peer review history

# Reviewer report: Reviewer response for version 2

**DOI:** 10.21956/wellcomeopenres.25186.r131590

**Published:** 2025-09-09

**Authors:** Nicky Wright

**Affiliations:** 1 Department of Psychology, Manchester Metropolitan University, Manchester, England, UK

I am happy with the edits made to the manuscript. I have one issue, that a 2014 reference is used to support a point about pandemic impact, this can not be correct? Please provide a reference on pandemic impact.

 Other studies have similarly found that younger children and boys are more likely to experience emotional difficulties during the pandemic (Randall *et al.*, 2014).

Is the work clearly and accurately presented and does it cite the current literature?

Partly

If applicable, is the statistical analysis and its interpretation appropriate?

Yes

Are all the source data underlying the results available to ensure full reproducibility?

Yes

Is the study design appropriate and is the work technically sound?

Yes

Are the conclusions drawn adequately supported by the results?

Partly

Are sufficient details of methods and analysis provided to allow replication by others?

Yes

Reviewer Expertise:

Developmental psychopathology

I confirm that I have read this submission and believe that I have an appropriate level of expertise to confirm that it is of an acceptable scientific standard.

# Reviewer report: Reviewer response for version 1

**DOI:** 10.21956/wellcomeopenres.22964.r79345

**Published:** 2024-05-09

**Authors:** Nicky Wright

**Affiliations:** 1 Department of Psychology, Manchester Metropolitan University, Manchester, England, UK

This study contains some interesting findings on the impact of the pandemic on a diverse sample of UK children. I have a few suggestions that I think would strengthen this manuscript:

 Abstract:

 In methods can you report the dates of the data from before the pandemic.

 Intro:

 The Paybus paper is previous publication from this sample, so it should be highlighted as such in the intro.

 Methods:

 The covid survey is described as including validated measures – are all the measures used in the analysis validated? Can that be made clear, and include the citation for activity profile (rather than readers have to check the cohort profiles).

 Results

 In the analysis of things that made the children happy, would it not make more sense to specifically reference family in the name of the second theme seeing as it refers to family

 You refer to table 2 as “We categorise any increase in difficulties score when children were found to experience more difficulties during the pandemic (compared to pre-pandemic) and these are shown in supplementary Table 2 available as *Extended data* ( Dickerson, 2024). This data was available for 749 individuals and n= 351 (46.9%) had an increase in SDQ score.” but table 2 has a title saying that the analysis is cross-sectional on 851?

 Discussion:

 Where does this % come from?: “Compared to a pre-pandemic measure of SDQ, parents reported that 12.9% of children developed difficulties in their social and emotional wellbeing during the pandemic”. In the results section you report that 8% of children who were not experiencing difficulties at the pre- experienced difficulties during the pandemic?

 This does not provide an explanation as to why boys may be more affected “ Younger children and boys might have experienced more negative impacts of the pandemic restrictions and/or anxieties relating to the virus itself, where older children may be better able to cope with the uncertainty.”

 In this sentence, state what the SDQ is more accurate than “Secondly, our study utilised the parent reported SDQ which may be better at reflecting accurately the child’s mental health. 

 Overall I think the discussion of the results needs to follow the two different analyses conducted. The bulk of the results section is showing associations between various factors and difficulties during the pandemic – but without accounting for pre-existing difficulties it is not known whether this reflects functioning prior to the pandemic or not. Less factors are associated with a change in difficulties, but it is important that the children living in the most deprived quintile were 3 times more likely to move above the threshold for experiencing difficulties (although this is not replicated in the analysis examining a change in continuous scores). When discussing the results, the distinction between what is associated with difficulties during the pandemic and with change should be made more clear. I would also make the point that most children did not experience a change (but that you did identify some factors associated with the change – with the caveat that this was only in the categorical analysis) and relate this to your prior publication by Paybus.

Is the work clearly and accurately presented and does it cite the current literature?

Partly

If applicable, is the statistical analysis and its interpretation appropriate?

Yes

Are all the source data underlying the results available to ensure full reproducibility?

Yes

Is the study design appropriate and is the work technically sound?

Yes

Are the conclusions drawn adequately supported by the results?

Partly

Are sufficient details of methods and analysis provided to allow replication by others?

Yes

Reviewer Expertise:

Developmental psychopathology

I confirm that I have read this submission and believe that I have an appropriate level of expertise to confirm that it is of an acceptable scientific standard.

# Response

**Published:** 2024-09-30

**Authors:** Rachael Moss

**Affiliations:** Born in Bradford, UK

**Nicky Wright, Department of Psychology, Manchester Metropolitan University, Manchester, England, UK (Approved)**

 This study contains some interesting findings on the impact of the pandemic on a diverse sample of UK children. I have a few suggestions that I think would strengthen this manuscript: **Abstract: **In methods can you report the dates of the data from before the pandemic. **Response: **We have now included the pre-pandemic dates in our abstract.  

**Intro: **The Paybus paper is previous publication from this sample, so it should be highlighted as such in the intro.

**Response:** We have now added a sentence to clarify this.  

**Methods:** The covid survey is described as including validated measures – are all the measures used in the analysis validated? Can that be made clear, and include the citation for activity profile (rather than readers have to check the cohort profiles).

**Response:** We have now added this detail to ensure this part is clear.  

**Results** In the analysis of things that made the children happy, would it not make more sense to specifically reference family in the name of the second theme seeing as it refers to family. **Response:** We have changed this in Table 7.  

 You refer to table 2 as “We categorise any increase in difficulties score when children were found to experience more difficulties during the pandemic (compared to pre-pandemic) and these are shown in supplementary Table 2 available as Extended data (Dickerson, 2024). This data was available for 749 individuals and n= 351 (46.9%) had an increase in SDQ score.” but table 2 has a title saying that the analysis is cross-sectional on 851?

**Response:** We have added an additional table of data here to aid clarity.

**Discussion:** Where does this % come from?: “Compared to a pre-pandemic measure of SDQ, parents reported that 12.9% of children developed difficulties in their social and emotional wellbeing during the pandemic”. In the results section you report that 8% of children who were not experiencing difficulties at the pre- experienced difficulties during the pandemic? This does not provide an explanation as to why boys may be more affected “ Younger children and boys might have experienced more negative impacts of the pandemic restrictions and/or anxieties relating to the virus itself, where older children may be better able to cope with the uncertainty.” In this sentence, state what the SDQ is more accurate than “Secondly, our study utilised the parent reported SDQ which may be better at reflecting accurately the child’s mental health.

**Response:** We have now re-worked our discussion to ensure it makes a distinction between cross-sectional analysis and SDQ change pre and post pandemic.  

 Overall I think the discussion of the results needs to follow the two different analyses conducted. The bulk of the results section is showing associations between various factors and difficulties during the pandemic – but without accounting for pre-existing difficulties it is not known whether this reflects functioning prior to the pandemic or not. Less factors are associated with a change in difficulties, but it is important that the children living in the most deprived quintile were 3 times more likely to move above the threshold for experiencing difficulties (although this is not replicated in the analysis examining a change in continuous scores). When discussing the results, the distinction between what is associated with difficulties during the pandemic and with change should be made more clear. I would also make the point that most children did not experience a change (but that you did identify some factors associated with the change – with the caveat that this was only in the categorical analysis) and relate this to your prior publication by Paybus.

**Response:** We have edited the discussion to make a distinction between cross-sectional associations and any change in experience in difficulties that we were able to observe.  

 Is the work clearly and accurately presented and does it cite the current literature? Partly

 Is the study design appropriate and is the work technically sound? Yes

 Are sufficient details of methods and analysis provided to allow replication by others? Yes

 If applicable, is the statistical analysis and its interpretation appropriate? Yes

 Are all the source data underlying the results available to ensure full reproducibility? Yes

 Are the conclusions drawn adequately supported by the results? Partly

# Reviewer report: Reviewer response for version 1

**DOI:** 10.21956/wellcomeopenres.22964.r79346

**Published:** 2024-05-03

**Authors:** Alison R McKinlay

**Affiliations:** 1 Research Department of Behavioural Science and Health, University College London, London, England, UK

Thank you for the opportunity to review this manuscript reporting data from the Born in Bradford study. The authors report on an important issue of children’s mental health and wellbeing, which was uniquely impacted during the COVID-19 pandemic relative to other age groups. The dataset provides a valuable opportunity to evaluate changes in children’s mental health and wellbeing in the early months of COVID-19 social distancing restrictions. It is an advantage and strength that the study has pre-pandemic cohort data to compare to during the pandemic.

 The Abstract section is clear but for the benefit of other readers, I think this could be slightly strengthened by including more specific details, such as the pre-pandemic data collection dates and exact age range of children in the survey. Also, the concluding Abstract statement is quite broad and this could be strengthened by highlighting potential policy implications or a call to action arising from the study findings.

 The introduction is clear and straightforward but ultimately missing some important contextual information that creates questions about the empirical foundations of the work:

1. The paper mentions no guiding theory, model or framework that informed the study rationale/design. This could be addressed perhaps by introducing the theoretical and/or philosophical underpinnings associated with the Born in Bradford study and/or citing the protocol paper earlier on than in the Methods section for other readers to refer to. More details on the mixed methods approach used during the study could also be added to the Methods to clarify the type of mixed methods design and approach to analysing the different forms of data.
2. As a reader, I’m not clear which “key vulnerabilities” are being investigated and why, so a little further rationale in the latter part of the Introduction could resolve these uncertainties.

A key concern is that the description of qualitative data analysis is too brief to allow for an appraisal by others. For instance, the process of coding has not been explained, nor the type of thematic analysis cited. No information on the quality of data analysis has been discussed (see Braun and Clarke, 2022, p.268-277 Ref [1]).

 More details regarding the qualitative data and results would aid interpretation and evaluation of the Methods and Results section. For instance, I can see that 808 participants completed the freetext questions; however, it is surprising that only two themes were generated from what might be considered quite a large qualitative dataset. Providing a range/mean of words from participants in their qualitative responses might help to clarify this. I also urge the authors to consider a slightly more descriptive theme label and remove/update the duplicated subtheme name of “Mental health” as this label does not tell the reader a lot about what was detailed in responses categorised under this theme/ subtheme. I would consider a more specific label if appropriate, such as “Experiences of mental health concerns.”

 In Table 1. the response categories for gender (boy/girl) are labelled as “sex” (male/female). Please refer to the Wellcome Open Research article guidelines for sex and gender equity in research.

 The finding that many participants expressed frequent reports of health anxiety (rather than “high levels” as I don’t believe this was objectively measured) is understandable and a really important consideration for those supporting children and young people in times of future public health emergencies. I think it would strengthen the Discussion around this key finding to contextualise this in terms of potential policy implications and development of future pandemic preparedness plans.

 The explanation that younger children may have found it more difficult to cope than older children makes sense but as there is no theoretical framework or explicit epistemological stance from the outset of the paper, the discussion here is not as detailed or comprehensive as it could have been. See suggestion regarding mention of appropriate theories/frameworks in the Introduction.

 Overall, I want to commend the authors for drawing attention to an important area of research with strong potential to contribute to the design of future pandemic preparedness plans. I look forward to seeing more of the work arising from the Born in Bradford and BiB COVID-19 research in the future.

Is the work clearly and accurately presented and does it cite the current literature?

Partly

If applicable, is the statistical analysis and its interpretation appropriate?

Yes

Are all the source data underlying the results available to ensure full reproducibility?

Yes

Is the study design appropriate and is the work technically sound?

Partly

Are the conclusions drawn adequately supported by the results?

Yes

Are sufficient details of methods and analysis provided to allow replication by others?

No

Reviewer Expertise:

Mental health and wellbeing during the COVID-19 pandemic; health inequality; health and clinical psychology; qualitative research methods

I confirm that I have read this submission and believe that I have an appropriate level of expertise to confirm that it is of an acceptable scientific standard, however I have significant reservations, as outlined above.

## References

[1] : Thematic analysis: A practical guide to understanding and doing (1st ed.). *SAGE Publications* .2021;

# Response

**Published:** 2024-09-30

**Authors:** Rachael Moss

**Affiliations:** Born in Bradford, UK

**Alison R McKinlay, Research Department of Behavioural Science and Health, University College London, London, England, UK (Approved with reservations)**

 Thank you for the opportunity to review this manuscript reporting data from the Born in Bradford study. The authors report on an important issue of children’s mental health and wellbeing, which was uniquely impacted during the COVID-19 pandemic relative to other age groups. The dataset provides a valuable opportunity to evaluate changes in children’s mental health and wellbeing in the early months of COVID-19 social distancing restrictions. It is an advantage and strength that the study has pre-pandemic cohort data to compare to during the pandemic. The Abstract section is clear but for the benefit of other readers, I think this could be slightly strengthened by including more specific details, such as the pre-pandemic data collection dates and exact age range of children in the survey. Also, the concluding Abstract statement is quite broad and this could be strengthened by highlighting potential policy implications or a call to action arising from the study findings.

**Response: **We have now added the pre-pandemic data collection dates in along with the age ranges of children in our survey. We have added more detail and strengthened our concluding statement.  

 The introduction is clear and straightforward but ultimately missing some important contextual information that creates questions about the empirical foundations of the work:

 The paper mentions no guiding theory, model or framework that informed the study rationale/design. This could be addressed perhaps by introducing the theoretical and/or philosophical underpinnings associated with the Born in Bradford study and/or citing the protocol paper earlier on than in the Methods section for other readers to refer to. More details on the mixed methods approach used during the study could also be added to the Methods to clarify the type of mixed methods design and approach to analysing the different forms of data.

**Response: **We have removed the phrase ‘mixed methods’ from the title, as on reflection and from suggestion from another reviewer we feel this study is not a true mixed methods study. We have additionally added detail in the methods section and we have cited our protocol paper earlier in the introduction too.

 As a reader, I’m not clear which “key vulnerabilities” are being investigated and why, so a little further rationale in the latter part of the Introduction could resolve these uncertainties.

**Response: **We have edited the introduction so that it now includes more information on co-variates to ensure it includes information about variables that are key to the Born in Bradford cohort.

 A key concern is that the description of qualitative data analysis is too brief to allow for an appraisal by others. For instance, the process of coding has not been explained, nor the type of thematic analysis cited. No information on the quality of data analysis has been discussed (see Braun and Clarke, 2022, p.268-277 Ref [1]). More details regarding the qualitative data and results would aid interpretation and evaluation of the Methods and Results section. For instance, I can see that 808 participants completed the freetext questions; however, it is surprising that only two themes were generated from what might be considered quite a large qualitative dataset. Providing a range/mean of words from participants in their qualitative responses might help to clarify this. I also urge the authors to consider a slightly more descriptive theme label and remove/update the duplicated subtheme name of “Mental health” as this label does not tell the reader a lot about what was detailed in responses categorised under this theme/ subtheme. I would consider a more specific label if appropriate, such as “Experiences of mental health concerns.”

**Response: **In order to aid clarity, we have now added additional detail in the qualitative ‘data analysis’ section and the ‘qualitative analysis’ results section. We hope this additional detail helps provide a better understanding of what we did and how we analysed this data.  

 In Table 1. the response categories for gender (boy/girl) are labelled as “sex” (male/female). Please refer to the Wellcome Open Research article guidelines for sex and gender equity in research.

**Response:** We have now altered this so that it refers to gender in table 1.  

 The finding that many participants expressed frequent reports of health anxiety (rather than “high levels” as I don’t believe this was objectively measured) is understandable and a really important consideration for those supporting children and young people in times of future public health emergencies. I think it would strengthen the Discussion around this key finding to contextualise this in terms of potential policy implications and development of future pandemic preparedness plans.

**Response: **We have discussed this in the discussion to help elaborate how we operationalised this level of health anxiety. We do not have any pre-pandemic data regarding worries to compare the children’s experience of worry.  

 The explanation that younger children may have found it more difficult to cope than older children makes sense but as there is no theoretical framework or explicit epistemological stance from the outset of the paper, the discussion here is not as detailed or comprehensive as it could have been. See suggestion regarding mention of appropriate theories/frameworks in the Introduction.

**Response: **We appreciate this comment. We have re-worked the introduction so believe that this is more comprehensive now.  

 Overall, I want to commend the authors for drawing attention to an important area of research with strong potential to contribute to the design of future pandemic preparedness plans. I look forward to seeing more of the work arising from the Born in Bradford and BiB COVID-19 research in the future.  

 Is the work clearly and accurately presented and does it cite the current literature? Partly

 Is the study design appropriate and is the work technically sound? Partly

 Are sufficient details of methods and analysis provided to allow replication by others? No

 If applicable, is the statistical analysis and its interpretation appropriate? Yes

 Are all the source data underlying the results available to ensure full reproducibility? Yes

 Are the conclusions drawn adequately supported by the results? Yes

# Reviewer report: Reviewer response for version 1

**DOI:** 10.21956/wellcomeopenres.22964.r76983

**Published:** 2024-04-22

**Authors:** Finiki Nearchou

**Affiliations:** 1 Department of Psychology, University College Dublin, Dublin, Ireland

Thank you for inviting me to review this paper. This is an interesting study on children’s emotional and behavioural well-being during the COVID-19 pandemic. The study employed a longitudinal design using quantitative and qualitative methods. The findings are interesting and have the potential to add to this field of research.

 One of my main comments is using the term ‘mixed methods’ study: A mixed methods study comprises a unified design where segments contribute to answer a research question. In my opinion, this paper presents qualitative and quantitative findings added together to answer distinct research questions. There is no information about the design, rationale, epistemological stance, level  and type of mixing, to justify a mixed methods research design. Please see the work of Creswell for more details. Hence, I believe that the terms ‘mixed methods’ should be removed from the title.

 Please see more detailed comments per section below.

 Introduction

 Although there is some discussion on the impact of the pandemic on health inequalities in children, this is very brief. There is a wealth of recent and emerging literature, including systematic reviews and meta-analyses, on this area from which authors can draw from to elaborate more in depth and present a more comprehensive landscape of inequalities especially related to ethnicity, low SES etc. in children during the pandemic.

 In addition, there is a wealth of evidence on the impact of the pandemic on children’s emotional and social well-being which should be presented and discussed by authors.

 Aim 3): Why did authors decide to include an investigation strand on children’s worries? Please justify.

 Methods Participants and Collection

 A short narrative on some study details will be helpful to appear in this paper too in addition to citing the paper’s protocol.

 Covariates:

 Physical activity and food insecurity: (See also my earlier comment in the introduction). It is unclear as to why these modifiable factors were included as covariates related to the impact of the COVID-19 pandemic on children’s behavioural difficulties. Authors need to elaborate and expand in the introduction using evidence to support their arguments.

 Results:

 ‘’The odds of White British children experiencing difficulties were two times that of Pakistani heritage children (OR 0.55, 95% CI 0.35-0.86).’’ This is unclear, something’s missing, please clarify.

 Discussion:

 The discussion at its current state is limited presenting only an interpretation of findings in relation to some existing literature. There is neither an in-depth elaboration nor a comprehensive discussion of the real-world implications of these findings. This study presents longitudinal findings and is one of the few available in the pre-post pandemic context. Implications should be  better situated within the national context but also placed within a more international context as well. What is the impact of this research, nationally and internationally? Is there any applicability of these findings in other contexts and in the light of a future pandemic or epidemic?

 Minor

 Please use past tense e.g. ‘ the aim of this paper was to..’ since the research has been completed. Please amend throughout.

Is the work clearly and accurately presented and does it cite the current literature?

Partly

If applicable, is the statistical analysis and its interpretation appropriate?

Yes

Are all the source data underlying the results available to ensure full reproducibility?

Yes

Is the study design appropriate and is the work technically sound?

Partly

Are the conclusions drawn adequately supported by the results?

Yes

Are sufficient details of methods and analysis provided to allow replication by others?

Yes

Reviewer Expertise:

youth mental health, research methods, clinical and health psychology, risk and resilience

I confirm that I have read this submission and believe that I have an appropriate level of expertise to confirm that it is of an acceptable scientific standard, however I have significant reservations, as outlined above.

# Response

**Published:** 2024-04-29

**Authors:** Rachael Moss

**Affiliations:** Born in Bradford, UK

Thank you for your time and comments on our paper Dr. Finiki Nearchou. We will be responding in detail after we have received our other peer review comments. Thank you.

# Response

**Published:** 2024-09-30

**Authors:** Rachael Moss

**Affiliations:** Born in Bradford, UK

**Finiki Nearchou, Department of Psychology, University College Dublin, Dublin, Ireland (Approved with reservations)**

 Thank you for inviting me to review this paper. This is an interesting study on children’s emotional and behavioural well-being during the COVID-19 pandemic. The study employed a longitudinal design using quantitative and qualitative methods. The findings are interesting and have the potential to add to this field of research. One of my main comments is using the term ‘mixed methods’ study: A mixed methods study comprises a unified design where segments contribute to answer a research question. In my opinion, this paper presents qualitative and quantitative findings added together to answer distinct research questions. There is no information about the design, rationale, epistemological stance, level  and type of mixing, to justify a mixed methods research design. Please see the work of Creswell for more details. Hence, I believe that the terms ‘mixed methods’ should be removed from the title. **Response:** Thank you for your comment. We understand your opinion on this. We have now removed ‘mixed methods’ from our title.

**Please see more detailed comments per section below.**

**Introduction**

 Although there is some discussion on the impact of the pandemic on health inequalities in children, this is very brief. There is a wealth of recent and emerging literature, including systematic reviews and meta-analyses, on this area from which authors can draw from to elaborate more in depth and present a more comprehensive landscape of inequalities especially related to ethnicity, low SES etc. in children during the pandemic. In addition, there is a wealth of evidence on the impact of the pandemic on children’s emotional and social well-being which should be presented and discussed by authors.

**Response: **We were aiming to be as concise as possible in our introduction, however, we admit we may have therefore omitted some more recent literature. We have therefore added some new text into the introduction that focuses on the impact the pandemic had for children (supported by more recent literature).  

**Aim 3):** Why did authors decide to include an investigation strand on children’s worries? Please justify.

**Response: **We have now added a section in the introduction which refers to the concept of worries and helps explain why we have chosen to focus on this.

**Methods Participants and Collection**

 A short narrative on some study details will be helpful to appear in this paper too in addition to citing the paper’s protocol.

**Response:** We have now added some additional detail in the ‘setting’ sub-section of this section to aid in clarity and understanding.

**Covariates:**

 Physical activity and food insecurity: (See also my earlier comment in the introduction). It is unclear as to why these modifiable factors were included as covariates related to the impact of the COVID-19 pandemic on children’s behavioural difficulties. Authors need to elaborate and expand in the introduction using evidence to support their arguments.

**Response:** Please see earlier comment above. We have now added additional information on this in our introduction.  

**Results: **‘’The odds of White British children experiencing difficulties were two times that of Pakistani heritage children (OR 0.55, 95% CI 0.35-0.86).’’ This is unclear, something’s missing, please clarify.

**Response: **We have amended this sentence so that it reads more clearly.  

**Discussion: **The discussion at its current state is limited presenting only an interpretation of findings in relation to some existing literature. There is neither an in-depth elaboration nor a comprehensive discussion of the real-world implications of these findings. This study presents longitudinal findings and is one of the few available in the pre-post pandemic context. Implications should be better situated within the national context but also placed within a more international context as well. What is the impact of this research, nationally and internationally? Is there any applicability of these findings in other contexts and in the light of a future pandemic or epidemic?

**Response:** We have now added some additional literature to the discussion focusing on alternative findings identified in other national and international studies using similar cohorts.  

**Minor**

 Please use past tense e.g. ‘ the aim of this paper was to..’ since the research has been completed. Please amend throughout.

**Response:** We have re-read our manuscript and made sure that the past tense is being used throughout.  

 Is the work clearly and accurately presented and does it cite the current literature? Partly

 Is the study design appropriate and is the work technically sound? Partly

 Are sufficient details of methods and analysis provided to allow replication by others? Yes

 If applicable, is the statistical analysis and its interpretation appropriate? Yes

 Are all the source data underlying the results available to ensure full reproducibility? Yes

 Are the conclusions drawn adequately supported by the results? Yes
